# Fast Skeletal Muscle Troponin Activator *tirasemtiv* Increases Muscle Function and Performance in the B6SJL-SOD1^G93A^ ALS Mouse Model

**DOI:** 10.1371/journal.pone.0096921

**Published:** 2014-05-07

**Authors:** Darren T. Hwee, Adam Kennedy, Julie Ryans, Alan J. Russell, Zhiheng Jia, Aaron C. Hinken, David J. Morgans, Fady I. Malik, Jeffrey R. Jasper

**Affiliations:** Cytokinetics, Inc., South San Francisco, California, United States of America; University of Edinburgh, United Kingdom

## Abstract

Amyotrophic Lateral Sclerosis (ALS) is a motor neuron disease characterized by progressive motor neuron loss resulting in muscle atrophy, declining muscle function, and eventual paralysis. Patients typically die from respiratory failure 3 to 5 years from the onset of symptoms. *Tirasemtiv* is a fast skeletal troponin activator that sensitizes the sarcomere to calcium; this mechanism of action amplifies the response of muscle to neuromuscular input producing greater force when nerve input is reduced. Here, we demonstrate that a single dose of *tirasemtiv* significantly increases submaximal isometric force, forelimb grip strength, grid hang time, and rotarod performance in a female transgenic mouse model (B6SJL-SOD1^G93A^) of ALS with functional deficits. Additionally, diaphragm force and tidal volume are significantly higher in *tirasemtiv*-treated female B6SJL-SOD1^G93A^ mice. These results support the potential of fast skeletal troponin activators to improve muscle function in neuromuscular diseases.

## Introduction

Amyotrophic Lateral Sclerosis (ALS) is a debilitating and fatal disease characterized by the selective and progressive loss of motor neurons in the spinal cord leading to atrophy, weakness, and eventually complete paralysis of skeletal muscle. ALS is the most common motor neuron disease in adults, approximately affecting 22,000 individuals in the United States alone [1]. Nearly 10% of ALS cases are familial, of which, approximately 20% are caused by dominantly inherited mutations in the Cu/Zn superoxide dismutase gene, SOD1 [2]. Transgenic mice carrying ALS-associated mutant human SOD1 genes, including the B6SJL-SOD1^G93A^ mouse, parallel many features of the human disease [3]. B6SJL-SOD1^G93A^ mice develop progressive limb and body weakness at approximately 80 days of age, culminating in full limb paralysis, morbidity and death at around 135–140 days [3]. Many of the histological features of disease in the B6SJL-SOD1^G93A^ mice are similar to those observed in ALS patients, although there appears to be less robust enlargement and sprouting of neighboring motor units towards denervated muscles as compensation for the loss of the primary motor neuron [4]. The only approved medication for ALS patients to date is riluzole, which potentially extends life an average of 2 to 3 months, and also extends lifespan in the B6SJL-SOD1^G93A^ mouse [5], [6]. However, riluzole has not been shown to improve muscle strength or respiratory function in ALS patients [7].

*Tirasemtiv* is novel small molecule activator of the fast skeletal muscle troponin complex. *Tirasemtiv* selectively sensitizes fast skeletal muscle troponin to calcium (Ca^2+^), and slows the rate of Ca^2+^ release from the regulatory troponin complex of fast skeletal muscle [8]. In intact skeletal muscle *in situ*, the compound amplifies the response of muscle to nerve input and increases force generation at sub-maximal levels of nerve stimulation. In the present studies, B6SJL-SOD1^G93A^ mice were treated with single doses of *tirasemtiv* to investigate its potential effects on skeletal muscle function *in* *vitro* and *in* *vivo*, including assessments of muscle strength and endurance as well as respiratory function. Our results demonstrate that *tirasemtiv* improves muscle strength in B6SJL-SOD1^G93A^ mice exhibiting functional deficits, and supports the hypothesis that *tirasemtiv* may benefit patients with ALS or other neuromuscular diseases that exhibit impaired neural input.

## Materials and Methods

### Ethics Statement

Animals used in this study were maintained in accordance with the *Guide for the Care and Use of Laboratory Animals of the Institute* (Seventh Edition, National Research Council) and under the supervision and approval of the Cytokinetics Institutional Animal Care and Use Committee.

### Transgenic Mice

Wild-type background strain B6SJLF1/J mice and B6SJL-SOD1^G93A^ mice over-expressing the human SOD-1 gene with mutation G93A were licensed from Northwestern University and received from Jackson Labs, Inc (strains 100012 and 002726, respectively; Bar Harbor, ME) at approximately seven weeks of age and monitored for signs of muscle weakness. All mice were group-housed in a 12-hour light cycle and fed standard chow (Lab Diet 5001) and water *ad libitum*. Body mass was recorded twice weekly. Grip strength, rotarod performance and grid hang-time was evaluated weekly. No more than one test was performed each day. Once the animal’s grip strength declined by 25% from baseline, full evaluation in the various functional assessments ensued following either vehicle or *tirasemtiv* administration. Female mice were used for motor performance tests due to their being less aggressive to cage mates and more easily trained to rotarod and hang test equipment in our hands. When grip strength declined by 25% from baseline mice were given Nutragel in addition to normal chow and water for the remainder of the study. Prior to each test interval, study personnel evaluated animals for general health, ambulation and condition of forepaws and hindlimbs. Mice were only evaluated assuming there was no evidence of impaired ambulation, discomfort or injuries to extremities, forepaw and footpad sensitivity or injuries from grip test assay. Mice were euthanized by CO_2_ asphyxiation, followed by exanguination, prior to reaching the point of severe limb weakness or paralysis.

### Functional Assessments of Muscle Performance

Baseline measurements of female mouse body weight, forelimb grip strength, grid hang-time and rotarod performance were recorded initially at ten weeks of age and weekly thereafter for the following eight weeks. Once forelimb grip strength declined by 25%, or by 40%, B6SJL-SOD1^G93A^ mice performed a battery of functional tests following vehicle or *tirasemtiv* treatment. *Tirasemtiv* or vehicle was administered by oral gavage (10 mg/kg dose in 0.5% HPMC/0.2% Tween 80) 30 minutes prior to each test (grip strength, hang time and rotarod). This dose was expected to provide a peak plasma concentration of approximately 3–4 µg/ml between 20–30 minutes after dosing. The investigator was blinded to treatment and the mice were given the same treatment on successive days, with each assessment occurring on a separate day. Mice were re-randomized to treatment between 25% and 40% test periods. Statistical significance was calculated by t-test between vehicle and treated group at specific animal age and p<0.05 was considered significant.

### Assessment of Grip Strength

Forelimb and hindlimb grip measurements were acquired in triplicate with a 250 gram Dual Sensor Grip Meter (DFS-R-250G, Transcat, Inc., Rochester, New York, USA). The mice were lowered onto a triangle bar of the grip strength meter until the animals gripped the bar with their forelimbs or hindlimbs, then the mice were pulled gently backward until they released their grip. The force gauge of the grip meter recorded the maximum force. Twelve female mice were used for each vehicle and *tirasemtiv* study group for grip strength tests.

### Grid Hang-time Assessment

A modified cage grid apparatus was designed to allow mice to grab the cage wire mesh grid and then the grid was inverted 180 degrees. The time before the mice dropped off of the grid on to a soft pad below was recorded for each assessment with a maximum time of 300 seconds. Only one series of tests was performed for each session and the animals were returned to their cages. The test was repeated weekly throughout the lifespan of the mice.

### Rotarod Performance Assessment

Mice were evaluated by placing them on the rotarod (San Diego Instruments, San Diego, CA) rotating drum (rod) and allowing them to run/climb at a low constant speed (12 RPM) for ten minutes during their initial training. Following the training period, animals were evaluated on the rotarod weekly to measure time to fall at 12 RPM. The maximum time for the assay was set at 10 minutes.

### Preparations of EDL Muscle for *in situ* Analysis

*In situ* muscle testing in a cohort of female mice occurred at two stages of disease: at 90–100 days of age, when signs of weakness were becoming apparent (weakness and trembling of hindlimbs) and a later stage, 110–115 days where signs of single or dual limb paralysis were evident. Mice were placed under anesthesia using isoflurane and the skin around the experimental leg was removed. The distal end of the EDL muscle and its associated tendon were then isolated. The mouse was placed on the platform of an Aurora *in situ* muscle analysis apparatus (model 806C; Aurora Scientific, Inc., Aurora, Ontario, Canada). Body temperature was maintained with a circulating water system. The knee was immobilized in a clamp between two sharpened screws and the distal tendon was cut and tied to the arm of a force transducer (Aurora Scientific, Ontario, Canada) using a silk suture. The muscle was stimulated via the peroneal nerve. For nerve isolation, a 0.5 cm incision was made at the upper thigh and the overlying biceps femoris muscle was cut to expose the peroneal nerve. The nerve was then dissected free of surrounding connective tissue and a pair of stainless steel needle electrodes (0.10 mm) were placed on the exposed nerve.

Muscle contractile properties were assessed by recording the force generated following stimulation of the peroneal nerve. Muscle length (L_o_) was adjusted to produce the maximum isometric force after sub-maximal stimulation (30 Hz, 1 ms pulses, 350 ms train duration). Once L_o_ had been established, the nerve was stimulated every 2 minutes at a selected frequency ranging from 10–200 Hz (1 ms pulses, 350 ms train duration). To measure relative changes in the force-frequency relationship following *tirasemtiv* dosing, the muscle was stimulated at 5 Hz for the course of the experiment. Twitch force was the force of a single stimulus-contraction-relaxation sequence measured at 10 Hz stimulus. Excessive tissue drying was alleviated by periodically dripping Krebs buffer (117.5 mM NaCl, 4.7 mM KCl, 1.2 mM KH_2_PO_4_, 1.18 mM MgSO_4_, 2.5 mM CaCl_2_, 25 mM NaHCO_3_, 11 mM glucose) onto the exposed muscle. This preparation was stable for 3–4 hours.

*Tirasemtiv* was administered in solution (50% PEG300/10% EtOH/40% Cavitron cyclodextrin formulation) as a single slow bolus over a 2 minute period via a catheter in the contralateral femoral artery placed above the aortic bifurcation. *Tirasemtiv* bolus injections (2, 2, 2, and 4 mg/kg) were given at approximately 20 min intervals to achieve a cumulative dose of 10 mg/kg in order to assess the dose response, with a maximal dosage volume of 5 ml/kg. At the end of each experiment, a single terminal blood sample was drawn via cardiac puncture for compound concentration analysis. Muscles were excised, the length and weight of the muscle measured, and the force was normalized to the physiological cross sectional area of the muscle (N/cm^2^, described in (8). Metrics measured from these experiments included stimulated and baseline tension.

### Diaphragm Muscle Tension *ex vivo*

Diaphragm contractile force was measured by electrical field stimulation in an organ bath system (Radnoti) based on a standard operating protocol adapted from the Treat NMD website.

(http://www.treat-nmd.eu/downloads/file/sops/dmd/MDX/DMD_M.1.2.002.pdf). The diaphragm and the last floating rib from WT and B6SJL-SOD1^G93A^ mice were excised, rinsed in physiological saline, and placed in a temperature controlled water-jacketed chamber (26–27°C) containing Krebs-Henseleit Buffer (118 mM NaCl, 10 mM glucose, 4.6 mM KCl, 1.2 mM KH_2_PO_4_, 1.2 mM MgSO_4_*7H_2_O, 24.8 mM NaHCO_3_, 2.5 mM CaCl_2_, 50 mg/L tubocurarine, 50U/L insulin, pH:7.4) that was continuously aerated with 95% O_2_/5% CO_2_. After 10 minutes of equilibration, vertical strips spanning the floating rib to the central tendon were cut from diaphragms. Braided silk sutures were tied at the central tendon and floating rib and attached to a force transducer between two platinum electrodes. Diaphragm strips were set to a length that produced maximum twitch tension (Lo). The force-frequency profile of the muscle was obtained by stimulating the muscle at frequencies between 10–150 Hz (Grass Stimulator, 0.6 pulse width, 800 ms train duration). *Tirasemtiv* was dissolved in DMSO (1 or 3 µM) and directly added into the bath.

### Whole Body Plethysmography to Evaluate Respiratory Function

WT and B6SJL-SOD1^G93A^ female mice were orally dosed with vehicle or 10 mg/kg *tirasemtiv* and placed in unrestrained whole body plethysmography chambers for 30 minutes of acclimation. After acclimation, respiratory parameters, including tidal volume, respiratory rate, and minute ventilation, were monitored for 10 minutes at room air. Upon completion of baseline room air measurements, animals were exposed to a 5% CO_2_ gas mixture for 30 minutes. After the 5% CO_2_ exposure, animals were re-exposed to room air and monitored.

### Chronic *tirasemtiv* Studies

Beginning at approximately nine weeks of age, B6SJL-SOD1^G93A^ transgenic mice were fed Harlan Teklad Rodent Diet 8604 chow containing *tirasemtiv* (400 ppm for females or 600 ppm for males), or control chow. The different doses were structured to achieve comparable plasma levels in the different sex mice. Mice were weighed and assessed for clinical signs once per week using a scoring system composed of three metrics: Gait, balance and ability to ambulate, each scored on a five point scale by the investigator during the course of the study. Once muscle weakness was evident, animals were weighed and scored more frequently based on the severity of weakness. Humane endpoints used to determine time of euthanasia included weight loss of greater than 20%, dual limb (hind limb or forelimb) paralysis or a low expectation of survival until the next time point. Investigators judging animal viability were blinded to whether or not animals were being treated with control chow or *tirasemtiv-*formulated chow. The median age of survival was calculated using Kaplan-Meier method for the vehicle control and *tirasemtiv*-treated mice respectively. A proportional hazard Cox regression model was used to calculate the hazards ratio between two treatment groups of mice in time to reaching the humane endpoint using vehicle as a control.

### Statistical Analyses

Statistics for performance and *in situ* studies were performed using students t-test and ANOVA via GraphPad Prism (La Jolla, Ca). Chronic-treatment survival studies were performed using the Kaplan-Meier method and a Cox regression model via SAS statistical software (SAS institute, Cary, NC).

## Results

### B6SJL-SOD1G93A Transgenic Mice Exhibit Deficits in Muscle Strength and Performance

**Figure 1:**
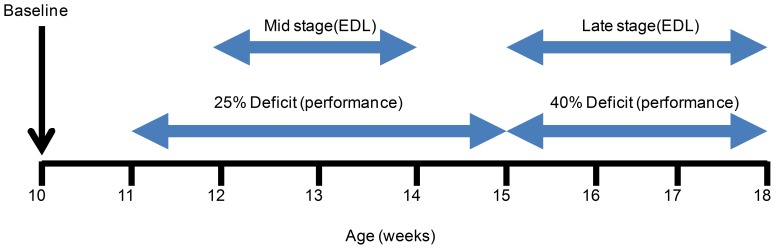
Timeline of performance assays and *in situ* EDL muscle force assessment as a function of B6SJL-SOD1^G93A^ mouse age. The mid-stage assessment of EDL *in situ* force measurements correlated with the timing of performance assays at a 25% forelimb grip strength deficit. Late stage assessment correlated with the timing of performance assays at 40% forelimb grip strength deficit. Unrestrained whole body plethysmography (uWBP) was performed after each surviving animal completed the final performance assays at approximately 18 weeks of age.

**Figure 2:**
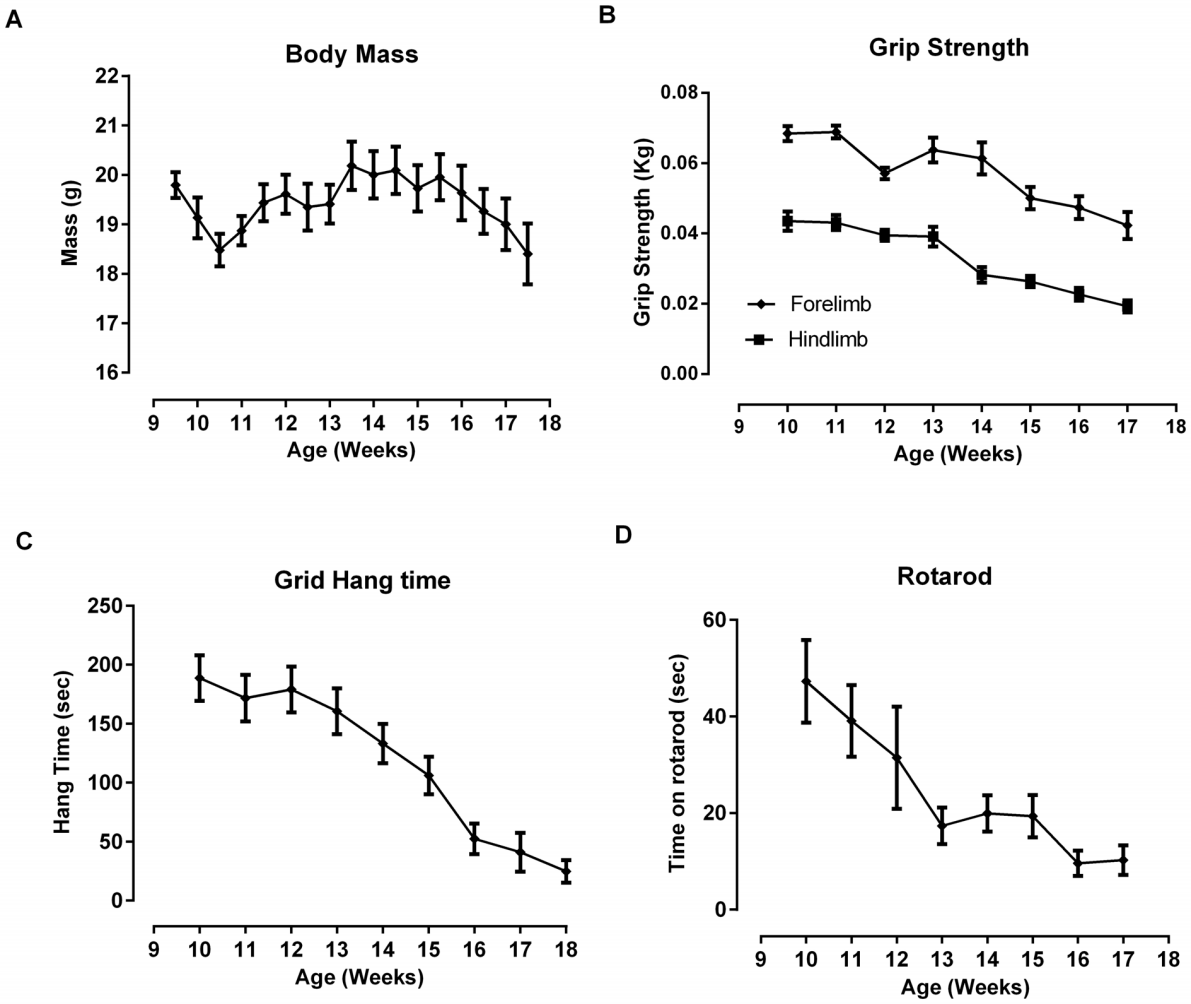
Body mass and muscle performance decreases over time in female B6SJLSOD1^G93A^ mice : A) Body mass, B) Forelimb and hind limb grip strength, C) Grid-hang time, and D) Rotarod performance over 10 to 18 weeks of age. Tests were performed on a weekly basis. Data from mice under assessment with either vehicle or *tirasemtiv* were excluded from these figures for that week. Data are expressed as mean ± SEM. n = 24/group.

**Figure 3:**
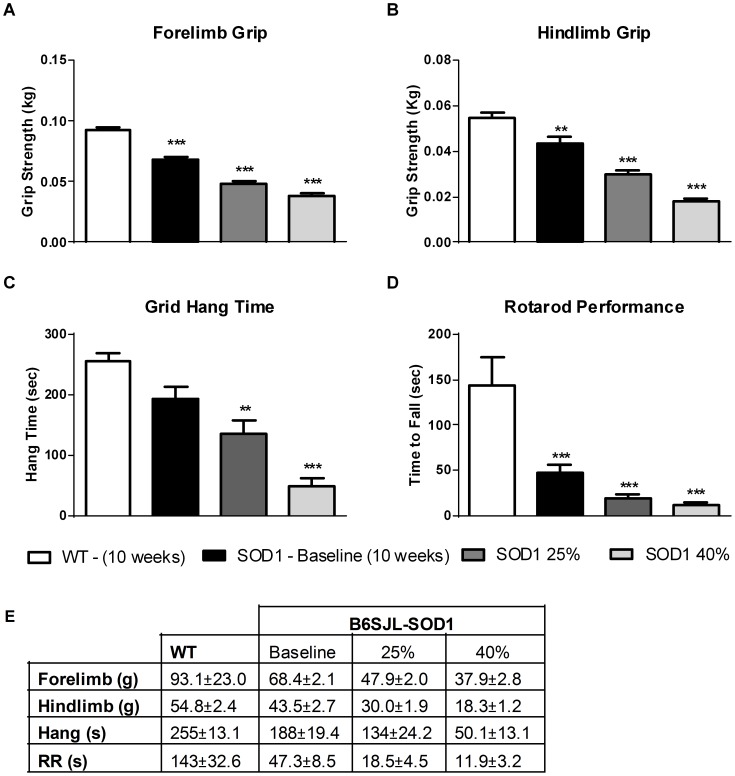
Female B6SJL-SOD1^G93A^ mice exhibit significant functional deficits prior to *tirasemtiv* administration. Performance values of (A) Forelimb grip strength, (B) Hindlimb grip strength, (C) Hang test performance, and (D) Rotarod performance at point of selection for 25% and 40% deficit milestones in forelimb grip compared to the 10-week old baseline. (E) Numerical values for WT, 10 week SOD1 baseline, 25% and 40% milestones. Data are expressed as mean ± SEM. n = 18–24/group. ** = p<0.01, *** = p<0.001 vs. WT by one way ANOVA with post-hoc Tukey’s test.

SOD1^G93A^ copy number influences the variability of disease progression in B6SJL-SOD1^G93A^ mice [9]. In addition to investigating the functional effect of *tirasemtiv* at time dependent mid- and late age disease points, animals were also treated and tested with a battery of functional assays upon demonstrating a 25% or 40% deficit in forelimb grip strength from baseline measurements made at ten weeks of age. Forelimb grip strength was chosen because it is a simple, reproducible assay with known deficits corresponding with the development of the disease phenotype in this model [10], [11]. Figure 1 shows the correlation, by age, of SOD1^G93A^ mice assessed at a 25% and 40% deficit alongside assessments of muscle strength in SOD1^G93A^ mice at a mid-stage and late-stage of the disease progression using *in situ* EDL assessments. Figure 2 illustrates the progression of disease in the four muscle performance assessments, illustrating a decline in all four assessments over time. Deficits in forelimb and hind limb grip strength correlated with the ability to hold onto a cage lid/grid while upside down (grid hang time) and the ability to remain on a rotating cylinder (rotarod). Functional performance in the weeks preceding the 25% (ranging from 11–15 weeks of age) and 40% (14–18 weeks of age) forelimb grip strength deficit revealed significant reductions in hindlimb strength (30% and 65% decrease from baseline), hang time (35% and 75% decrease) and rotarod time to fall (60% and 75% decrease), respectively (Figure 3).

### Single doses of *tirasemtiv* Increase Muscle Performance in Moderately Weak B6SJL-SOD1G93A Mice

**Figure 4:**
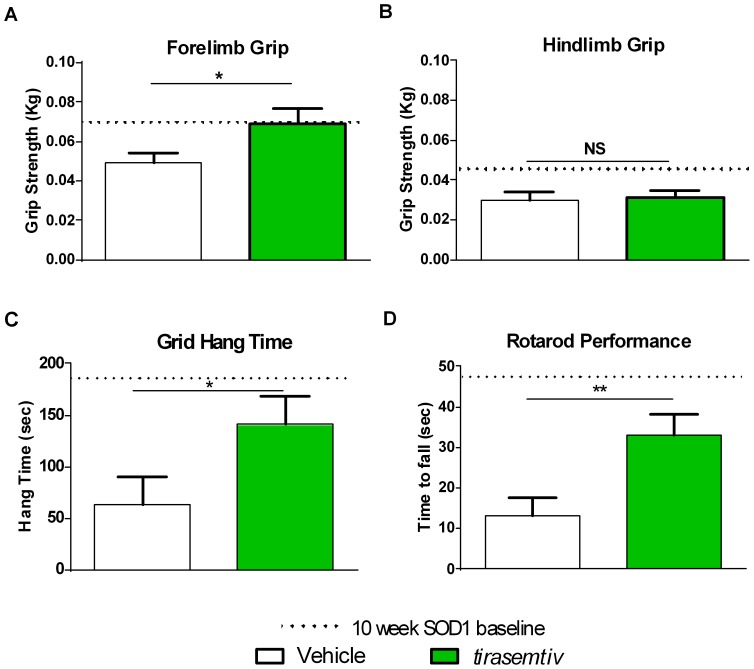
*Tirasemtiv* increases forelimb grip strength, grid hang time, and rotarod performance in female B6SJL-SOD1^G93A^ mice. Effect of *tirasemtiv* at 25% (approx. 90–110 days) forelimb grip strength loss milestone compared to 10-week old baseline on (A) Forelimb grip, (B) Hindlimb grip, (C) Grid hang time, and (D) Rotarod performance. Dotted line shows mean B6SJL-SOD1^G93A^ transgenic mouse performance assay values at baseline (10 weeks of age). Data are expressed as mean ±SEM. n = 10–12/group. * = p<0.05, ** = p<0.01 vs. WT by one way t-test.

At the 25% deficit milestone, vehicle-treated B6SJL-SOD1^G93A^ mice demonstrated forelimb grip strength of 49.6±4.6 g. *Tirasemtiv* increased grip strength by 38% to 68.6±8.1g (p<0.05, single tailed t-test) (Figure 4A). By the 40% deficit milestone, *tirasemtiv* did not increase grip strength (non-significant by single-tailed t-test). No change in hind limb grip strength by *tirasemtiv* was observed at either 25% (Figure 4B) or 40% of deficit (not shown).

At the 25% forelimb grip strength deficit milestone, vehicle-treated grid hang time performance (Figure 4C) was 62.7±27.6s, which was 75% below baseline grid hang time. *Tirasemtiv* treatment increased grid hang time by 125% to 140.8±27.3s (P<0.01 single tailed t-test). Grid hang test performance deteriorated rapidly between the 25% and 40% deficit assessments, with a hang time of 15±7.7s in vehicle-treated B6SJL-SOD1^G93A^ mice at the 40% deficit milestone. *Tirasemtiv* did not improve hang time at the 40% deficit (not shown).

Vehicle-treated B6SJL-SOD1^G93A^ at the 25% grip strength deficit milestone had a rotarod performance (Figure 4D) time of 13.1±4.3s, a 72% decrease from the ten week baseline. *Tirasemtiv* increased the time to fall by 150% to 33.1±5.0s (p<0.01 single tailed t-test). Like grid hang time, rotarod performance declined rapidly, with transgenic mice unable to perform on the rotarod at the 40% grip strength deficit milestone (not shown).

### *Tirasemtiv* Increases Sub-maximal Muscle Tension in Moderately Weak B6SJL-SOD1^G93A^ Mice

**Figure 5:**
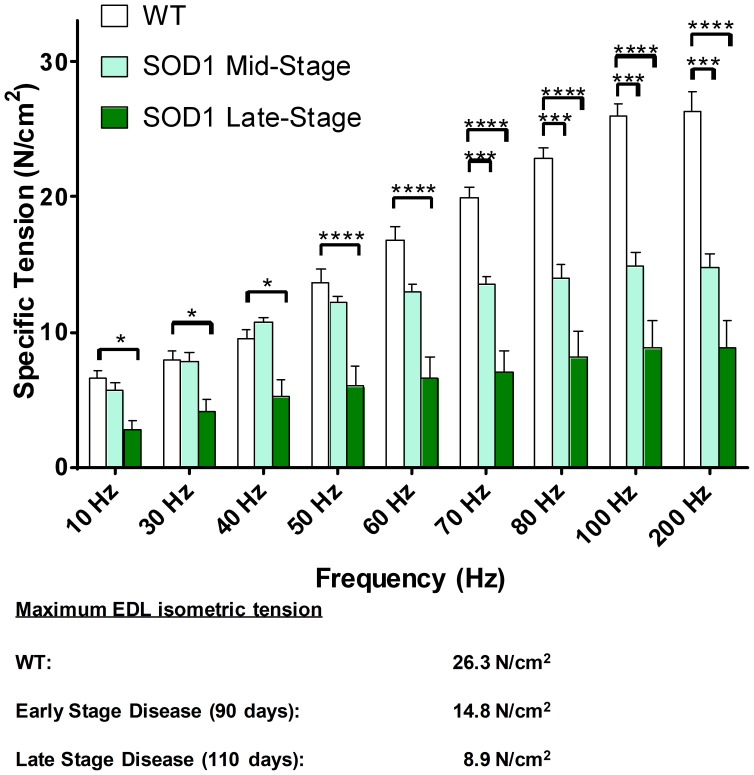
Isometric *in situ* force is reduced with age in B6SJL-SOD1^G93A^ mice. *In situ* assessment of EDL isometric force-frequency relationship in WT and B6SJL-SOD1^G93A^ transgenic mice at two stages of progressive neuromuscular disease. EDL muscle tension was assessed at mid-stage of disease (age = 90–100 days) when signs of physical weakness were just apparent and late-stage disease (110–115 days) when frank limb paralysis was becoming evident using an *in situ* preparation of the extensor digitorum longus (EDL) muscle. (B6SJL mice, n = 8; mid-stage B6SJL-SOD1^G93A^ mice, n = 5; late-stage B6SJL-SOD1^G93A^ mice, n = 6; Data are expressed as mean specific tension ± SEM. *p<0.05, ***p<0.001, ****p<0.0001 vs. WT).

In a separate cohort of B6SJL-SOD1^G93A^ mice, isometric muscle force output was assessed at two time points: a ‘mid’ stage (age = 90–100 days) when signs of physical weakness were just apparent and a ‘late’ stage (110–115 days) when frank limb paralysis was becoming evident. Similar assessments were made in age-matched B6SJL/F1 wild-type (WT) mice as controls. The force-frequency relationship was measured by stimulating the EDL muscle via the peroneal nerve *in situ* over a range of frequencies from 10 to 200 Hz (Figure 5). Maximal tetanic force (200 Hz stimulation) was significantly reduced by 44% (14.8±1.0 N/cm^2^ (mean ± SEM), p<0.0001) in mid-stage and 66% (8.9±2.0 N/cm^2^, p<0.0001) in late-stage B6SJL-SOD1^G93A^ mice compared to WT mice measured between 90–100 days of age (26.3±1.6 N/cm^2^), respectively, reflecting a progressive loss of muscle strength with disease progression. In contrast to maximal tetanic force, twitch force (10 Hz stimulation; WT 6.58±0.54 N/cm^2^, n = 8) declined later in the course of the disease, with a 14% loss (5.75±0.58 N/cm^2^, n = 6, p = 0.34) in mid-stage and 57% loss (2.82±0.58 N/cm^2^, n = 7, p = 0.0004) in late-stage B6SJL-SOD1^G93A^ mice.

**Figure 6:**
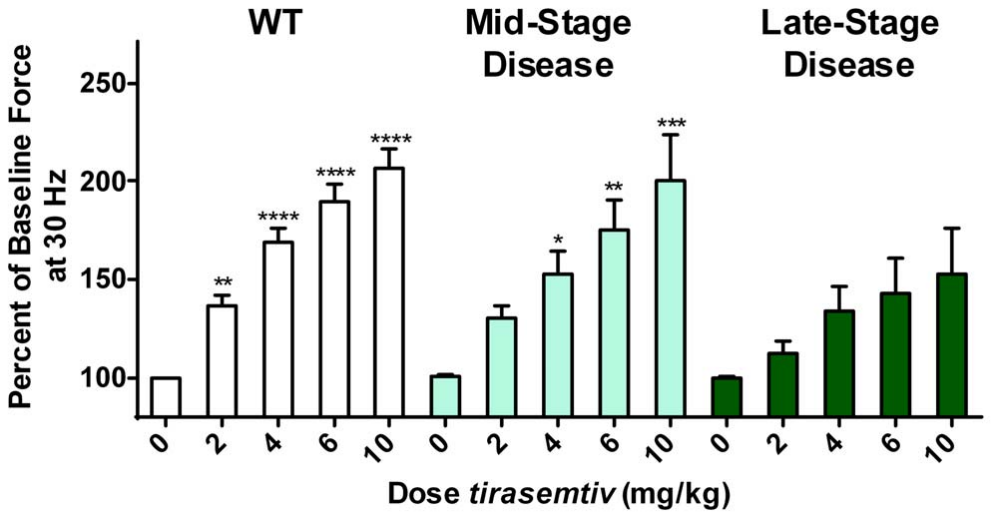
Infusion of *tirasemtiv* increases *in situ* EDL muscle force in wild-type and B6SJL-SOD1^G93A^ transgenic mice at mid-stage (90–100 days) and late stage (110–115 days) disease. The EDL muscle was stimulated every 2 minutes with a 5(1 ms pulse width, 350 ms train duration) via the peroneal nerve. *Tirasemtiv* was then administered as a 2 minute i.v. bolus in four cumulative doses up to a total of 10 mg/kg. (WT mice, n = 8; mid-stage B6SJL-SOD1^G93A^ mice, n = 5; late-stage B6SJL-SOD1^G93A^ mice, n = 6 Data are expressed as mean tension ± SEM. *p<0.05, **p<0.01, ****p<0.0001 vs. respective baseline force).

Isometric muscle force (sub-tetanic, 30 Hz stimulation) rapidly increased in a dose-dependent fashion following additive i.v. *tirasemtiv* doses of 2, 2, 2, and 4 mg/kg given at approximately 20 min intervals to achieve a cumulative dose of 10 mg/kg (Figure 6). Following *tirasemtiv* treatment, the specific tension of the WT mice increased from 6.41±0.29 N/cm^2^ to 12.3±0.42 N/cm^2^ at the top of the cumulative dose curve. In mid-stage B6SJL-SOD1^G93A^ mice the specific EDL muscle tension increased from 5.81±0.46 N/cm^2^ to 10.8±0.71 N/cm^2^, and in late-stage mice specific EDL tension increased from 3.34±0.73 N/cm^2^ to 5.05±1.4 N/cm^2^. Regression analysis of the log dose vs. response relationship indicated that *tirasemtiv* significantly increased muscle force in WT and mid-stage B6SJL-SOD1^G93A^ mice (WT p<0.0001; mid-stage p = 0.0028). At later stages of disease, the mice exhibited a trend for increased muscle function in response to *tirasemtiv* compared to baseline (p = 0.064). Terminal plasma compound levels following the 10 mg/kg *tirasemtiv* dose were not different between any of the groups.

### *Tirasemtiv* Increases Tidal Volume in Female B6SJL-SOD1^G93A^ Mice

**Figure 7:**
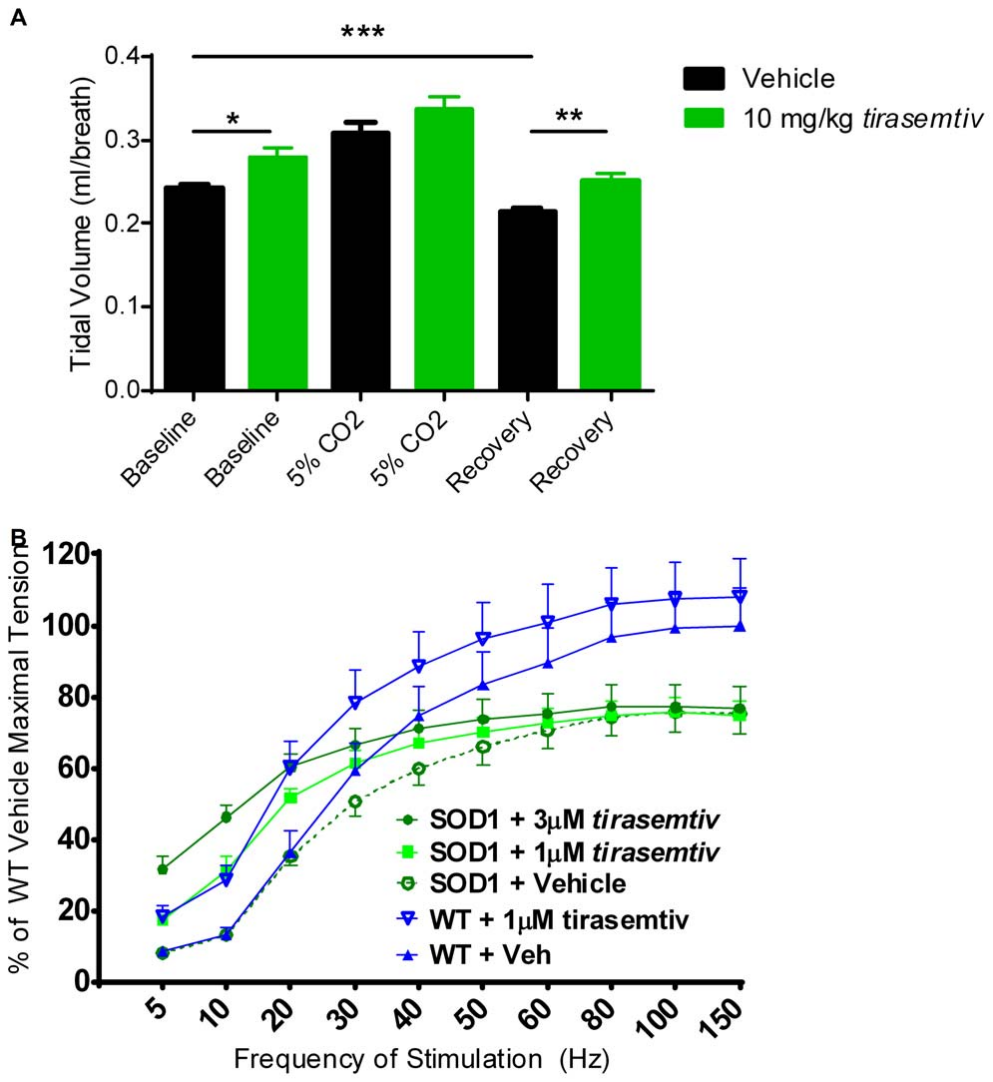
*Tirasemtiv* increases tidal volume and submaximal diaphragm tension in late stage female B6SJL-SOD1^G93A^ mice. Respiratory function and diaphragm tension output were assessed in B6SJL-SOD1^G93A^ mice at 18 weeks of age. (A) B6SJL-SOD1^G93A^ mice dosed with *tirasemtiv* had significantly higher tidal volume than vehicle-dosed mice at baseline and during recovery following a 5% CO_2_ exposure. Compared to its baseline tidal volume, vehicle-treated B6SJL-SOD1^G93A^ mice had significantly lower tidal volume during recovery. (B) Harvested WT and B6SJL-SOD1^G93A^ diaphragms treated with *tirasemtiv* produced greater tension in response to submaximal frequencies of electrical stimulation. Data are expressed as mean ± SEM. n ≥5/group.

Respiratory failure is a major cause of death in ALS patients, and decreases in tidal volume are noted with disease progression [12], [13]. The respiratory parameters of vehicle and *tirasemtiv*-treated WT and B6SJL-SOD1^G93A^ mice were assessed by unrestrained whole body plethysmography before, during, and after a 30 minute exposure to a 5% CO_2_ environment. Compared to vehicle-treatment, *tirasemtiv* (10 mg/kg) significantly increased tidal volume in B6SJL-SOD1^G93A^ mice exposed to room air (Figure 7A). Both vehicle and *tirasemtiv*-treated B6SJL-SOD1^G93A^ mice increased tidal volume in response to 5% CO_2_. Once the 5% CO_2_ stressor was removed, tidal volume was significantly below pre-CO_2_ normoxic exposure in vehicle-treated B6SJL-SOD1^G93A^ mice (p<0.001, by t-test). *Tirasemtiv*-treated B6SJL-SOD1^G93A^ mice maintained a significantly higher tidal volume than vehicle-treated B6SJL-SOD1^G93A^ mice during the 10 minutes of normoxic air respiration post-CO_2_ exposure.

### *Tirasemtiv* Increases Sub-maximal Tension in Isolated Diaphragm Tissue from Female B6SJL-SOD1^G93A^ Mice

Phrenic motor neuron loss and diaphragm atrophy has previously been observed in SOD1 mice [14]. In this study, diaphragm muscle strips were harvested from WT and B6SJL-SOD1^G93A^ mice and subjected to electrical field stimulation in a tissue bath set-up. Within respective WT and B6SJL-SOD1^G93A^ transgenic groups, *tirasemtiv* (1 µM, 3 µM) significantly increased tension compared to vehicle-treated diaphragm strips up to 20Hz stimulation of the muscle (Figure 7B). Of note, at stimulation frequencies between 5 to 30 Hz, *tirasemtiv* increased B6SJL-SOD1^G93A^ transgenic diaphragmatic tension development to levels even higher than WT diaphragm muscle. In vehicle-treated tissues there was a trend (p values between 0.05 and 0.10) toward lower tension production in B6SJL-SOD1^G93A^ transgenic diaphragms compared to WT tissue at stimulation frequencies greater than 50 Hz, suggesting diminished maximal tension development in B6SJL-SOD1^G93A^ transgenic mice.

### Chronic *tirasemtiv* Treatment in B6SJL-SOD1^G93A^ Mice does not Negatively Affect Lifespan

**Figure 8:**
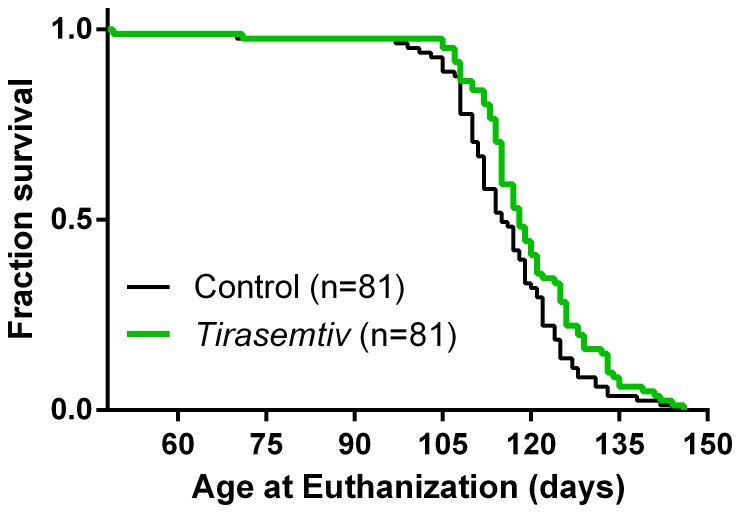
Chronic *tirasemtiv* exposure had no deleterious effect, and slightly increases survival time of B6SJL-SOD1^G93A^ mice. Male and female B6SJL-SOD1^G93A^ transgenic mice fed chow containing *tirasemtiv* (400 ppm for females or 600 ppm for males) beginning at nine weeks of age survived longer to a humane endpoint compared to control chow-fed mice (p = 0.009; hazard ratio = 0.66). Data presented were pooled from blinded studies conducted in B6SJL-SOD1^G93A^ mice randomized to *tirasemtiv* or control chow (n = 81 female and male mice treated with *tirasemtiv*; n = 81 control female and male mice).

A separate cohort of B6SJL-SOD1G93A mice fed control chow or chow containing *tirasemtiv* from approximately nine weeks of age to death (or humane endpoint) examined potential effects from chronic treatment. A log-rank analysis stratified for cohort differences showed no deleterious effect of chronic *tirasemtiv* exposure; similarly a recent study in dy2J muscular dystrophy mice treated chronically with *tirasemtiv* did not result in harmful effects in viability [15]. The median age of survival for the vehicle control and *tirasemtiv*-treated mice was 115 and 118 days, respectively. A post-hoc analysis of pooled data from six test groups of B6SJL-SOD1G93A mice revealed a modest prolongation in time to reaching the humane endpoint for *tirasemtiv*-treated mice compared to control chow-fed mice (Figure 8; p = 0.009; hazard ratio = 0.66).

## Discussion

The etiology of human ALS is unknown in approximately 80–90% of the cases, which presents a challenge for the development of treatments to mitigate or prevent disease progression. Thus, translating promising pre-clinical therapeutic data from ALS animal models into clinical efficacy has been hampered by the unknown pathophysiology, genetics, and mechanism(s) responsible for ALS [16]. The small molecule, fast skeletal muscle troponin activator, *tirasemtiv* (formerly CK-2017357), amplifies the contractile response of muscle to a given level of neural input. Here we demonstrate that *tirasemtiv* significantly improves muscle function in B6SJL-SOD1^G93A^ mice with progressive motor neuron deterioration. *Tirasemtiv’s* mechanism of action is not dependent on the specific disease mechanism for ALS, perhaps improving the odds of translation of these results into patients and more broadly, into other diseases of neuromuscular origin [8]. An exploratory study of possible pharmacodynamic responses to *tirasemtiv* revealed potential improvements in maximum ventilatory volume, a measure of diaphragm function, and submaximal handgrip dynamometer performance in ALS patients [17], possibly reflecting the pre-clinical results presented here.

SOD1 gene copy number and background strain affect the onset and rate of phenotypic ALS progression in various mouse models [9]. In consideration of this variability, the timing of *tirasemtiv* assessment in B6SJL-SOD1^ G93A^ transgenic mice was based on the functional decline of forelimb grip strength (either 25% or 40% deficit from initial assessment) rather than age of the mice. Female B6SJL-SOD1^ G93A^ transgenic mice used in this study reached a 25% forelimb grip strength deficit over a range of 77–105 days of age, and a 40% deficit between 98–126 days of age, illustrating the variability in disease progression by age. *Tirasemtiv* improved functional performance in B6SJL-SOD1^ G93A^ mice exhibiting a 25% functional deficit in grip strength. In particular, grid hang time (2.25x increase) and rotarod performance (2.5x increase) demonstrated the most striking improvements, suggesting that *tirasemtiv* may have a greater effect on dynamic activities that involve multiple muscle groups (Figure 4). In concordance with these functional data, *tirasemtiv* increased *in situ* isometric force of fast skeletal EDL muscle at submaximal nerve stimulation frequencies in B6SJL-SOD1^ G93A^ mice that were clearly weaker than their matched controls, supporting the hypothesis that *tirasemtiv* amplifies the response of muscle under conditions of reduced neural input [8].

At approximately the 40% deficit mark for forelimb grip strength (110–115 days), *in situ* EDL force assessment in B6SJL-SOD1^G93A^ mice revealed increased variability of the muscle force output in response to peroneal nerve stimulation. At this point in the disease course of the mice, it is important to note that some mice exhibited clinical signs of hindlimb paralysis despite the lack of forelimb paralysis. Previous studies have chronicled the earlier and more rapid decline in hindlimb motor unit number and muscle force as compared to forelimb motor unit number [4]. In contrast to the earlier age point assessment, *tirasemtiv* increased EDL force to a smaller extent at this stage of disease progression. B6SJL-SOD1^G93A^ mice at this stage typically did not demonstrate an improvement in the conscious functional assays following treatment with *tirasemtiv.* Not surprisingly, the effect of *tirasemtiv* diminishes as neuromuscular input diminishes and of course, no effect is expected in the absence of neuromuscular input. Collectively, the effect of *tirasemtiv* on muscle force generation was greater earlier in the course of the disease when frank paralysis was not present.

Respiratory failure stemming from progressive phrenic motor neuron deterioration and diaphragm atrophy is the primary cause of death in ALS patients [18]. Symptoms of respiratory weakness typically do not manifest until the latter stages of disease progression [19]. In this study, respiratory function and diaphragm muscle tension was investigated after female B6SJL-SOD1^G93A^ mice reached a 40% deficit in forelimb grip strength, or approximately 18 weeks of age. Consistent with prior work [20] respiratory function did not appear to be compromised in B6SJL-SOD1^G93A^ mice at this stage in their disease progression since breath frequency and tidal volume were not significantly different from age-matched WT mice at room air or when exposed to a 5% hypercapnic challenge (data not shown). Treatment with *tirasemtiv* did lead to decreased breath frequency and a significant increase in tidal volume (Figure 7A) which are indicative of an effect on diaphragm function. Consistent with its mechanism of action, *tirasemtiv* significantly amplified the response of diaphragmatic muscle to submaximal stimulation frequencies. This change in the sensitivity of muscle to neuromuscular input, as well as a decrease in muscle fatigability as observed in tests of peripheral muscle function, could underlie the effect on respiratory function in the B6SJL-SOD1^G93A^ mice. These preclinical data support the notion that *tirasemtiv* might improve respiratory mechanics in patients with ALS as is reported in recent clinical studies of *tirasemtiv* in ALS patients [17].

Recent muscle-targeted therapies in ALS mouse models have demonstrated significant improvement in muscle function, but no increase in lifespan [21], [22]. For example, although muscle-specific over-expression of IGF-1 maintained skeletal muscle integrity and protected motor neurons in the SOD1^G93A^ ALS mouse model [23],IGF-1 therapy was not beneficial to ALS patients in a two year trial [24]. The effect of *tirasemtiv* treatment on lifespan was not a primary objective of the current investigation; however, post-hoc analysis of pooled data from separate cohorts of B6SJL-SOD1^G93A^ mice fed chow containing *tirasemtiv* (from nine weeks of age) revealed a modest, but significant prolongation in time to reaching humane endpoint, increasing the median age of survival from 115 to 118 days (Figure 8). Given the poor translation of other, larger improvements in longevity to the human population, it is unlikely that this is indicative of *tirasemtiv* actually extending life, but is a clear indication that chronic administration of *tirasemtiv* over several weeks does not negatively impact survival, which we believe is an important observation. Since the compound for this particular study was administered in food, it was not possible to accurately assess the effects of *tirasemtiv* on aspects of disease progression, other than longevity, since *tirasemtiv* is only able to modify muscle function acutely and food intake is highly variable.

Most skeletal muscles operate at submaximal activation during routine activities [25]. The therapeutic hypothesis underlying the investigation of clinical effectiveness of *tirasemtiv* in ALS patients is that by directly increasing the submaximal force and power output of muscle and by increasing its resistance to fatigue, *tirasemtiv* will improve ALS patients’ ability to function in their daily lives. The current preclinical findings support the hypothesis that *tirasemtiv* may benefit patients with ALS or other neuromuscular diseases by increasing submaximal force generation in the face of reduced neuromuscular input.

## Supporting Information

Checklist S1 Prisma Checklist.
(DOC)
